# 

**Published:** 1900-12

**Authors:** 


### OUR SUCCESS IN THE HOUSE.

### MARRIAGE.

At Middleburgh, Pa., November 15, 1900, Dr. Clinton F. Keiter, of Elizabethville, Pa., of the class of 1899, Veterinary Department, University of Pennsylvania, to Miss Laura A. Smith, daughter of Mr. and Mrs. James P. Smith.

### ASSOCIATION MEETINGS.

A meeting of the South Dakota State Veterinary Medical Association will be held at Station Hotel, Huron, South Dakota, January 15, 1901.

The Iowa State Veterinary Medical Association will hold their annual meeting in Des Moines, January 23 and 24, 1901.

### PERSONALS.

Dr. Charles A. Dohan, of Darling, Pa., was one of the riders at the Thanksgiving Day exercises of the Farmers' Club of Philadelphia.

Dr. H. D. Fennimore, of Knoxville, Tenn., retired from veterinary practice on November 1st. He thinks the field offers a good opening for a young man.

Dr. H. L. B. Coote, formerly of Cheyenne, Wyoming, has located at Chicago, Ill.

Dr. W. F. Heyde, of St. Louis, Mo., has been suffering from an infected right hand—from septic infection.

Dr. H. D. Gill, of New York City, was the winner of many brushes with fast ones in October on the "Speedway" behind "Rockdale Boy," whose sale was confirmed at a good round figure early in November.

Veterinarians of the National Horseshow, Madison Square Garden, New York, were Drs. Wm. Sheppard, T. G. Sherwood, and John M. Heard.

Dr. E. M. Ranck, of Philadelphia, has joined the list of active supporters of building associations.

Dr. W. E. Orr, of the Bitter Root Stock Farm of the late Marcus Daly, had an experience coming East with a consignment of horses to the Madison Square Garden sale that he will remember for a long time. One of the horses under his care became seriously ill with nervous symptoms of a severe character, producing a frenzied condition, which Dr. Orr had to deal with in the car alone.

Dr. R. S. Huidekoper spent several days in Philadelphia in the latter part of November.

State Veterinarian Pearson, of Philadelphia, Pa., was an attendant at the fat-cattle show in Pittsburg early in November.

Dr. B. F. Bachman, of Strasburg, has removed to Pittsburg, Pa., where he will continue in the practice of veterinary medicine.

Early in November could be noted walking down Broad Street, Philadelphia, Drs. Huidekoper, Pearson, and Hoskins, earnestly in conference over army legislation prospects.

Dr. H. D. Gill, of New York City, was a winner of a blue ribbon at the National Horseshow in New York City in November in the trotting-bred class, with his well-known mare, "Rosalie," winning over such a famous blue-ribboner in this class as "Altamont."

Dr. Leonard Pearson, of Philadelphia, was a visitor to the "Smoky City" early in November.

Dr. Thomas Castor, not Charles Castor, was elected to membership in the A. V. M. A. in September. Dr. Castor is a graduate of the Veterinary Department, University of Pennsylvania, class of 1894. He is now located at Las Vegas, New Mexico, as an inspector of sheep under the Bureau of Animal Industry.

Dr. W. H. Dalrymple, of Baton Rouge, La., has just returned from Europe.

Dr. A. F. Schrieber, of Philadelphia, takes an active interest in the food-show held annually in the "Quaker City." Someone was unkind enough to say that he was a fair representative of what Mrs. Rorer's cooking does for her subjects.

Dr. J. P. Turner, of Washington, D. C., was a visitor to Philadelphia in November on leave of absence.

Dr. Harry D. Martin, of Buffalo, N. Y., was a late visitor to Atlantic City and Philadelphia in October.

Dr. H. A. Paget, of Scranton, Pa., has returned from his European tour much improved from his impaired physical condition.

Dr. Lawrence A. Nolan, of Baltimore, was elected secretary of the Bryan-Stevenson and Nicholas W. Steele Democratic Club at its organization early in October.

Dr. A. G. Hopkins, of the Wisconsin Agricultural Experiment Station, is a frequent contributor to *The Farmer's Advocate*, published at London, Ontario.

Dr. Eugene Battin, of the United States Quartermaster's Department, and recently located at Manila, P. I., is on a furlough to the States, and spent a portion of his time in and about Philadelphia in October.

Dr. John J. Maher, of Philadelphia, maintains an attractive dog-ambulance in his practice.

Dr. J. G. Annand, of Minneapolis, Minn., has been charged with the field-work of hog-cholera in that State under the direction of the State Veterinarian.

Dr. C. J. Marshall was veterinarian at the show of the Philadelphia Kennel Club in November.

Dr. A. T. Peters, of Lincoln, Neb., is spending many hours among the farmers at the Institutes held throughout the State, enlightening them on the work of the Veterinary Department of the State Experiment Station.

Dr. W. S. Kooker, of Philadelphia, has been in poor health for some time, at times unable to care for his practice.

### INDEX.

ABORTION, review of contagious, 151
Abortive treatment of boils, 690
Absorption, has the bladder any power of? 162
Acarus-mange in dogs, 420
Acetanilid in epilepsy, 690
Acetic acid, fluid extracts made with, 689
Acoin, a formula for the hypodermatic injection of, 162
Actinomycosis in dogs, 421
Action of certain somnifacients on the horse, 193, 278
of the thyroid gland, 689
Added strength, an, 699
Address to veterinary profession of New Jersey, 148
Adulteration of cocoa leaves, 295
of yellow wax, 298
Ailments, observations on the foot and its, 13
Alcohol, sterilization of catgut by, 551
Aloes varieties, distinct therapeutic properties of, 688
Alverson, A. G., V.S., 168
American Public Health Association, 514, 576
*versus* European inspection, 140
Veterinary College, 442
Medical Association, 179, 236, 308, 438, 442, 511, 633, 711, 775
book review, 176
at Detroit, 428, 633, 775
executive committee, 1900–1901, 711
Among new books and publications, 47
the boards of examiners, 576
the colleges, 648
those who hinder progress, 358
Anæsthetic purposes, paraldehyde and chloroform for, 623
Analysis, urinary, in veterinary practice, 608
Ancient use of strychnine, 551
Andre, V. J., V.S., 470
Aneurism in a chicken, case of, 228
Angora goats, strongylus contortus in a herd of, 423
Animals and man, relation of tuberculosis in domesticated, 465
communicable diseases of domestic, that materially affect the livestock industry, 134
experimental tuberculosis, human and bovine, in the domestic, 715
losses of farm, 223
rheumatism in the domestic, 65
State work with infectious diseases of, 155, 268, 333
Annales de Médecine Veterinaire, 159
Annual report of corresponding secretary of Pennsylvania State Veterinary Medical Association, 246
Another bureau loss, 235
field of usefulness, 508
in the fold, 565
Anthrax, action of creolin in, 158
treated with hypodermatic injections of carbolic acid, 689
Antidote, borax as a general poison, 626
camphorated oil a carbolic acid, 167
to strychnine, salt an, 488
Antifebrine in infectious pneumonia, 687
Antisepsis in surgery, practical, 615
Antiseptic treatment of wounds, new publications, 48
therapeutics, 649, 744
wound oil, 487
Antistreptococcic serum, 297
Antitetanic serum as a prophylactic, 297
Antitoxin treatment of distemper in horses, the, 171
Apomorphine counteracted by boric acid, 626
Apoplexy, parturient, a discussion of milk-fever, 671
under Schmidt's treatment, 286
Are we growing too fast? 632
Army, the veterinary corps of, 41
legislation, 174, 490
report of committee on, 569
legislative committee appeal, A. V. M. A., 236
should have a veterinary corps, why the, 299
veterinary corps, 305, 361, 493, 552
service, 566
Arsenic in organic bodies, 487
Asepsis and antisepsis, routine manipulations and operations from the stand-point of, 1, 82
Aspergillus fumigatus, a case of pneumonomycosis due to the, 451
Association meetings, 252, 778
news, 177
usefulness, 632
Atlantic City in 1901, 564, 699
A. V. M. A.:
Thirty-seventh Annual Convention at Detroit, 428, 633, 775
Azoturia, lead as a possible cause of, 488

BACILLI, note on examination of milk for tubercle bacilli, 395
Bacillus of Koch into the milk of a tuberculous woman, passage of the, 353
Balch, Dr. A. W., 488
Belladonna, 198
Bender, Harry E., secretary, 61, 188
Bichloride eyewash, 487
Bitch, prolapsus of the uterus in the, 95
of the vaginæ in the, 414
Bitting, A. W., 646
Berliner Thieraerzt Wochenschrift, 159
Bladder any power of absorption, has the, 162
Blood, Harvey and the circulation of the, 298
the rôle of iron in forming, 162
Boards of examiners, 441, 576
Bodies, arsenic in organic, 487
Boils, abortive treatment of, 690
Book reviews, 47, 176, 701, 774
Borax as a general poison antidote, 626
Boric acid, apomorphine counteracted by, 626
Bottle-loving cat, 546
Bovine species, a prodromic symptom of rabies in the, 158
Bowels in the cow, digestive stasis of the, 342
Bradley, Horace L., V.S., secretary, 60
Bradley, John L., V. S., 232
Brenton, S., 512
Brimhall, S. D., V.M.D., 722
Brown, S. D., V. S., 575

Bureau of Animal Industry, the, 220
improvements in, 568
notes, 700
loss, another, 235
Burkholder, S. G., M.D., V.S., 7, 88
Burns, potassium chlorate for, 231
Butterfield, J. F., V.S., 23

CALCULI, in the dog, urethral, 205
Calves, an experiment in the transmission of syphilis to, 264
Campbell, W. T., D.V.S., 96, 341
Camphor, production of, 486
Camphoroxol, 232
Canine and feline medicine and surgery, department of, 292, 350, 414, 543, 631, 691
Canine and Feline Surgery, Hobday, book review, 701
Canine-hopples, the Dawson-French, 631
Carbolic acid, anthrax treated with hypodermatic injections of, 689
antidote, camphorated oil a, 167
in equine tetanus, large doses of, 624
Carcinoma in cattle, on, 385
Carpipes unilateralis congenitalis, 692
et luxatio capiti radii (congenitalis), 543
Case of helminthic migration, an extraordinary, 292
of open-joint where fluid had escaped, cure of a, 96
of rabies, report of, 93
reports, 39
Cases, some everyday, 37
Castration of cryptorchids (ridglings), 470
of an elephant, 478
Cat, a bottle-loving, 546
necrosis of the ulna, with fistula in a, 292
nymphomania in a castrated female, 414
Catgut, sterilization of, by alcohol, 551
Cattle at an altitude of 7400 feet, an outbreak of tuberculosis among, 407
carcinoma in, 385
hemorrhagic septicæmia iu, 732
in Montana, psoroptic scabies, 583
post-mortem inspection of, 217
traffic, obstacle to enforcing regulations requiring the tuberculin test in interState, 590, 675
Central west in line, the, 562
Charge, a serious, 234
Chicago Veterinary College, commencement exercises, 514
among the colleges, 648
Chicken, a case of aneurism in, 228
Chloretone, the effects and dangers of, 624
Chloroform for anæsthetic purposes, paraldehyde and, 623
Circulation of the blood, Harvey and the, 298
Clancy, Jos. B., secretary, 186, 254
Clark, W. G., secretary, 190, 250
Clement, A. W., 571
Clinical diagnosis of nasal discharges, 35
reports, 97
value of a leucocyte count in the diagnosis of septic infections, 12
Cocaine, hypnotics antagonistic to, 624
Cocoa leaves, adulteration of, 295
Colleges, among the, 648
Commencement exercises:
Chicago Veterinary College, 514
Grand Rapids Veterinary College, 310
Kansas City Veterinary College, 311
McKillip Veterinary College, 315
New York American Veterinary College, 433
New York State Veterinary College, Cornell University, 435
Ontario Veterinary College, 34, 312
United States College of Veterinary Surgeons, 435
University of Pennsylvania, Veterinary Department, 432
Commercial enterprise *vs.* paternalism, 175
Committee on army legislation, report of, 569
Communicable diseases of domestic animals that materially affect the livestock industry, 134
Communications, 50, 103, 237
Compound fracture of the tibia and fibula in a black and tan terrier, 691
Conditions reversed, 545
Congress, French veterinary, 1900, 186, 429
Conkey, L. L., dean, 428
Constitution and by-laws, A. V. M. A., proposed amendment to, 511
Contagious abortion, review of, 151
Control of glanders in Minnesota, State, 27
work, 238, 306
Convention, Detroit, September, 1900, A. V. M. A., thirty-seventh annual, 633
side lights of the Erie, 646
Cornell University, New York State Veterinary College, commencement exercises, 435
Correction, 633
Correspondence, 424, 490, 764
Country almost cleared of salable horses, 283
Covert, James R., 437
Cow, digestive stasis of the bowels in the, 342
Cows, an unclassified disease following parturition in, 24
Creolin in anthrax, action of, 158
soap, 488
Cribbing and roaring, resection of the spinal accessory nerve for, 99
Crowforth, Anderson, secretary, 253, 514
Current thoughts, 62

DALRYMPLE, W. H., M.R.C.V.S., 260, 328
Danger in using spider webs as a hæmostatic, 296
Dangers of an impure meat-supply and milk-supply, some of the, 260, 328
Dawson-French canine-hopples, 631
Defective eyelids, 401
Dehorning fluids, 164
Delaware, Richard R. Kenney, U. S. Senator from, 517
Department of canine and feline medicine and surgery:
Carpipes unilateralis congentalis, 692
et luxatio capiti radii (cougenitalis), 543
Cat, a bottle-loving, 546
Compound fractures of the tibia and fibula in a black and tan terrier, 691
Conditions reversed, 545
Dawson-French canine-hopples, 631
Dentition of a Mexican hairless dog, 693
Dog, acute indigestion in a, 646
peculiar case of functional derangement of the skin in a, 350
Duodenum, neoplasm of the, 293
Hæmatoma of the spleen, 292
Helminthic migration, an extraordinary case of, 292
Luxatio femoris in a lion, 415
Maternal instinct, an unusual exhibition of the, 693
Necrosis of the ulna with fistula in a cat, 293
Nymphomania in a castrated female cat, 415
Peculiar case of functional derangement of the skin in a dog, manifested by hemorrhages and abnormal secretions, followed by death from acute infection, 350
Prolapsus vaginæ in the bitch, 414
Department of surgery:
Clinical diagnosis of nasal discharges, 35
Hoof diseases, 547, 627, 682, 756
Penis and sheath, 416
Surgery of the penis, 481
Departure, a new, 174

Detroit, A. V. M. A., 428, 633, 755
September, 1900, 45
Detroit's triumph, the central west in line, 562
Diagnosis of rabies, the rapid, 404
of septic infection, the clinical value of the leucocyte count in the, 129
Diarrhœa, tannoform in, 420
Digestive stasis of the bowels in the cow, 342
Dinwiddie, R. R., M.D., V.S., 528, 715
Diphtheria antitoxic globulins, 688
Discharges, the clinical diagnosis of nasal, 35
Discolored eserine solutions, 167
Disease, naftalan in obstinate skin, 166
Diseases common to man and animals, 731
Diseases, hoof, 547, 627, 682
of animals, State work with, 155, 268, 333
of domestic animals that materially affect the livestock industry, communicable, 134
of poultry, new publications, 177
Disinfection of the skin and hands, 165
Distemper or shipping fever in horses, the antitoxin treatment of, 171
Dodds, Dr. W. G. King, 478
Dog, acute indigestion in a, 546
mumps in the, 476
peculiar case of functional derangement of the skin in a, 350
the dentition of a Mexican hairless, 693
urethral calculi in the, 205
Dogs, acarus-mange in, 420
actinomycosis and strongylus gigas in, 421
Donaldson, T. A., V.S., 20
Dourine and its parasite, 411
Dressing, europhen as a surgical, 551
Duodenum, neoplasm of the, 293

EDITORIALS, 41, 101, 174, 234, 299, 358, 428, 493, 552, 632, 695
Added strength, an, 699
American Veterinary Medical Association at Detroit, 428
Among those who hinder progress, 358
Another field of usefulness, 508
Are we growing too fast? 632
Army legislation, 174
veterinary corps, 299, 305, 493, 552
Association usefulness, 632
Atlantic City in 1901, 564, 699
Bureau loss, another, 235
Central west in line, the, 562
Charge, a serious, 234
Commercial enterprise *vs* paternalism, 175
Correction, 633
Departure, a new, 174
Detroit, September, 1900, 45
Detroit's triumph, 562
Era, a newer and better, 175
French Veterinary Congress of 1900, 429
Give rabies and hydrophobia a rest, 359
Guard, the passing of the old, 563
Harvard veterinary department's peril, 771
History repeats itself, 303
Hour of our greatest need, the, 234
Iowa's need, 103
Michigan as a recruiting field, 429
Michigan's peril, 102
ways, 304, 510
Minnesota's loss, Iowa's gain, 509
New Jersey's triumph, 101
opportunity, 772
New York Quarantine Station, our, 47
Our success in the House, 773
Rabies and hydrophobia a rest, give, 359
Recognition, a well-deserved, 46
Serious charge, a, 234
Veterinary corps, U. S. Army, the, 41, 405
education, 769
Vice-presidents, what shall we do with our, 562
Editorials:
When will reason and judgment prevail? 695
Where in 1901? 428
Why the army should have a veterinary corps, 299
Education of lawmakers, 539
Effects and dangers of chloretone, 624
Elementary obstetrics, 737
Elephant, castration of an, 478
Ellis, Robert W., D.V.S., 318
Epicarin, 225
Equine tetanus, large doses of carbolic acid in, 624
Era, a newer and better, 175
Erie Convention, side lights of the, 646
Errata, 305
Eserine solutions, discolored, 167
Examination of milk for tubercle bacilli, note on, 395
Examiners, among the boards of, 576
Examining and licensing stallions for the stud, 536
Experimental tuberculosis, human and bovine, in the domestic animals, 715
Experiment in the transmission of syphilis to calves, 264
Europhen as a surgical dressing, 551
External wounds, healing of, 396
Extracts from foreign journals (French)—
Anthrax, the action of creolin in, 158
Dourine and its parasite, 411
Mumps in the dog, 476
Passage of the bacillus of Koch into the milk of a tuberculous woman, 353
Rabies in the bovine species, a prodromic symptom of, 158
Rabiform symptoms due to spiroptera, 355
Eyelids, defective, 401
Eyewash, bichloride, 487

FARM animals, losses of, 223
Felton, Howard B., B.S., V.M.D., 205
Fever, parturient apoplexy, a discussion of milk, 671
Field of usefulness, another, 508
Fish, James G., M.D.C., 217
Pierre A., D.Sc., D.V.M., 608
Fistula, ilio-rectal, 227
in a cat, necrosis of the ulna with, 292
Fistulæ, a new treatment for, 341
Fluid extracts, tinctures made from, 163
Fluids, dehorning, 164
Fœtal skeleton, retention of, 761
Foot and its ailments, observations on the, 13
Forage poisoning of horses, a preliminary report upon (so-called spinal meningitis), 654
Formaldehyde, solutions of, 687
Formula for the hypodermatic injection of acoin, a, 162
Foster, J. P., secretary, 710
French canine hoppies, the Dawson-, 631
Cecil, D.V.S., 292, 350, 414, 543, 631, 691
Veterinary Congress of 1900, 186, 429
Fuller, George S., V.S., 171.
Fumigatus, a case of pneumonomycosis due to the aspergillus, 451

GARNIER, M., 352
Gelbert, C. S., V.M.D., 95
Genesee Valley Veterinary Medical Association, 183
Germany, hog inspection in, 51
Gibson, J. I., V.S., 536
Gland, action of the thyroid, 689
Glanders in Minnesota, State control of, 27
Globulins, diphtheria antitoxic, 688
Goat, the, 443
Goats, strongylus contortus in a herd of Angora, 423

Golindrinera, or euphorbia prostata, for snake and insect bites, 165
Gould, fourth revised edition of, book review, 176
Gruber's reaction in hog cholera, 528
Guard, the passing of the old, 563
Guide to Practical Meat Inspection, new publications, 49

HÆMATOMA of the spleen, 292
Hæmostatic, danger in using spider webs as a, 296
Hammond, E. W., D.V.S., 395
Hands, disinfection of the skin and, 165
Harger, S. J. J., V.M.D., 97
Hartwig, A. H., M.D.C., 142
Harvey and the circulation of the blood, 298
Head, George Douglass, B. S., M.D., 129
Healing of external wounds, 396
 ointment, 487
 powder, 551
Health, the relation of veterinary medicine to the public, 532
Heart, suture wounds of, 230
Helmer, Jacob, 109
Helminthic migration, an extraordinary case of, 292
Hemorrhagic septicæmia in cattle, 722
Hermetic sealing of wounds, the immediate, 230
Historical pharmacy, 298
History ever repeats itself, 303
Hobday's Canine and Feline Surgery, book review, 701
Hog cholera, Gruber's reaction in, 528
 inspection in Germany, 51
Hogs, yellow, 480
Hoof diseases, 547, 627, 682, 756
Hoopes, Herbert, V.M.D., 342
Hopkins, A. G., V.S.. 172, 277
Hopples, the Dawson-French canine, 631
Horse, the action of certain somnifacients on the, 193, 278
 sarcoptic scabies of the, 583
Horses, country almost cleared of salable, 283
 forage poisoning of, a preliminary report upon, 654
 preliminary report upon forage poisoning of, 654
 the antitoxin treatment of distemper or shipping fever in, 171
Horseshoeing, 214, 325
Hoskins, W. Horace, secretary, 185, 571, 646
Hour of our greatest need, 234
Howe, W. E., V.S., D.V.M., 75
Huidekoper, Rush S., 237, 492, 501, 512, 517, 571
Hydrophobia, rabies and, 597, 663
Hypnotics antagonistic to cocaine, 624
Hypodermatic injection of acoin, a formula for the, 162
 injections of carbolic acid, anthrax treated with, 689

ILIO-RECTAL fistula, 227
Illinois State Veterinary Medical Association, 56, 711
 Veterinary Medical and Surgical Association, 184, 573
Improvements in the Bureau of Animal Industry, 568
Indiana Association of Veterinary Graduates, 120
Indigestion in a dog, acute, 546
Infectious diseases of animals, State work with, 155, 268, 333
 pneumonia, antifebrine in, 687
Influenza, 336
Inoculation against plague, 167
Insecticides, 165
Instruction, a plea for broader tendencies in veterinary, 521
Inter-State cattle traffic, obstacles to enforcing regulations requiring the tuberculin test, 590, 675
Iodine, age of tincture of, 295
Iowa's loss, Minnesota's gain, 509
 need, 103
 State veterinary board, 442
 veterinary law, 240
Iron in forming blood, the rôle of, 162

JAUNDICE, verminous, 480
Jay, Robert, M.D V , 35, 481, 547, 682, 756
Jobson, George, D.V.S., 385
Johnson, G. A., D.V.M., 615
Jopling, William, 105, 657

KANSAS City Veterinary College, commencement exercises, 311
Kaupp, B. F., D.V S., 711
Kean, T. J., V.M.D., 214
Kenney, Richard R., U. S. Senator from Delaware, 517
Keystone Veterinary Medical Association, 55, 252
Knight, E., sec., 184
Knowles, M. E., D.V.S., 583

LAKE, W. H., V.S., 473
Lambs, stomach worm in, 277
Lassar's sublimate, carbolic salve, 487
Lawmakers, education of, 539
Lead as a possible cause of azoturia, 488
Leech, G. Ed., M.D.C., 209, 273
Legislation, 178, 240
 army, 490
Leucocyte count in the diagnosis of septic infections, the clinical value of a, 129
Lewis, Henry D., 120
Licensing stallions for the stud, examining and, 537
Lion, luxatio femoris in a, 414
Lockwood, S., Secretary, 123, 382
Loeb, Leo, M.D., 385
Lowe, J. Payne, D.V.S., 321
Lowe, Wm. Herbert, D.V.S., 1, 82, 148, 532, 646
Luxatio femoris in a lion, 414
Luzader, I. M., V.S., 19

MAINE Veterinary Medical Association, 61, 189
Malarial parasite, quinine and the, 231
Manual of prescription writing and dosology, new publications, 49
Manipulations and operations from the standpoint of asepsis and antisepsis, routine, 1
Manitoba, the Veterinary Association of, 188
Marriages, 235, 305, 431
Martin, W. J., M.D.C., 161, 230, 295, 418, 485, 551, 623, 687, 711
Maryland State Board of Veterinary Medical Examiners, 441
Massachusetts Veterinary Association, 118
Masseters, spasm of the, 169
Maternal instinct, an unusual exhibition of the, 693
Mathews, E., 741
McCarthy, D. J., M.D., 404
McClure, S. W., V.M D., 407
McKenzie, K. J., sec.-treas., 125, 573
McKillip Veterinary College, 315, 648
Meat inspection, 7, 88, 237
 Guide to Practical, book review, 49
 of swine, post-mortem, 75
 -supply and milk-supply, some of the dangers of an impure, 250, 328
Median neurectomy, my experience with, 321
Medicine, vanadium as a, 232
Merillat, L. A., V.S., 35, 198
Metacarpo-phalangeal sheath, tendo-vaginitis of the, 344

Mexican hairless dog, the dentition of a, 693
Michener, J. Curtis, president, 109
Michigan as a recruiting field, 429
  State Veterinary Board, 441, 576
Michigan's peril, 102
  ways, 304, 510
Milk- and meat-inspection, municipal, 71
  fever, parturient apoplexy, a discussion of, 671
  for tubercle bacilli, note on examination of, 395
  of thyroidectomized goats, treatment of Basedow's disease by the, 418
Minnesota, State control of glanders in, 27
  State Veterinary Medical Association, 123, 572
Minnesota's loss, Iowa's gain, 509
Missouri Valley Veterinary Association, 111, 179, 250, 440, 712
  Veterinary Medical Association, 59, 575, 710
Montana, psoroptic scabies of cattle in, 583
Moore, R. C., D.V.S., 401
Morot, Ch., 187
Moyle, T. F., V.S., 737
Muir, E Stanton, Ph.G., V.M.D., 193, 278
Mule, the, 443
Mumps in the dog, 476
Municipal milk- and meat-inspection, 71
  meat-inspection, 257

NAFTALAN in obstinate skin disease, 166
Nasal discharges, the clinical diagnosis of, 35
Necrology—
  Heath, E. M., M.D.C., 360
  Jopling, Mrs. Wm., 431
  Mackey, Chas. A., V.S., 431
  Pearson, John A., V.M.D., 511
  Tegtmier, A., V.S., 360
Necrosis of the ulna, with fistula, in a cat, 292
Nelson, S. B., D.V M., 465
Neoplasm of the duodenum, 292
Neson, G. E., D.V.M., 39
Netherton, E. J., V.S., 71
Neurectomy, my experience with median, 321
  sciatic, 98
  zereform in, 418
Newby, T. B., V.S., 145
New Jersey, a call from, 712
    address to the veterinary profession of, 148
    veterinary medical association of, 121, 252, 381, 711, 712
New Jersey's triumph, 101
  York American Veterinary College, commencement exercises, 433
    County Veterinary Medical Association of, 317
    quarantine station, our, 47
    State Veterinary College, Cornell University, commencement exercises, 435
        Board, 576
        Medical Society, 441
New publications, 774
Niagara and Orleans County Veterinary Medical Society, 253, 514
Nighbert, J. D., V.S., 169
North Dakota State Board of Veterinary Medical Examiners, 441
Note on examination of milk for tubercle bacilli, 395
Notes, 51, 105, 226, 285, 442, 475, 582, 622
Nymphomania in a castrated female cat, 415

OBSERVATIONS on the foot and its ailments, 13
Obstacles to enforcing regulations requiring the tuberculin test in inter-State traffic, 590, 675
Obstetrics, elementary, 737
Œstrus ovis, 23
Oil, antiseptic wound, 487
  of turpentine as a therapeutic agent, 485
Ointment, healing, 487
Ontario Veterinary Association, 109
    College, 648
      commencement exercises, 34, 312
Ophthalmia, 209, 273
  periodic, 19
  verminous (intra-ocular filariasis), 172
Organic bodies, arsenic in, 487
Orleans County Veterinary Medical Society, Niagara and, 253, 514
Outbreak of tuberculosis among cattle at an altitude of 7400 feet, 407
Ox, psoroptic scabies of the, 586

PAINT (lead poisoning) in cows, 344, 761
Paraldehyde and chloroform for anæsthetic purposes, 623
Parasite, dourine and its, 411
  quinine and the malarial, 231
Parker, John M., D.V.S., 731
Parker, Joseph M., D.V.S., 441, 539
Parturient apoplexy, a discussion of "milk fever," 671
    under Schmidt's treatment, 286
  paresis, 142
    Schmidt's treatment of, 168
Parturition in cows, an unclassified disease following, 24
Passage of the bacillus of Koch into the milk of a tuberculous woman, 353
Pearson, Leonard, B. S., V.M.D., 51, 451, 521, 654
Penis and sheath, 416
  surgery of the, 481
Pennsylvania State Board of Veterinary Medical Examiners, 185, 423
    Live-stock Sanitary Board, 430
    Veterinary Medical Association, 58, 107, 243, 382, 703
  Veterinary Department, University of, commencement exercises, 432
    Medical Society, University of, 60, 187
Periodic ophthalmia, 19
Personals, 62, 125, 190, 253, 318, 382, 447, 515, 577, 713, 784
Peters, A. T., D.V.M., 140
Peters, Austin, M.R.C.V.S., 512, 590, 646, 675
Pharmacopœia, the Swiss, 690
  revision of the United States, 161
Phillips, Walker S., V. S., 37, 743
Plague, inoculation against, 167
Plaster of oxide of zinc, a, 419
  -of-Paris, hard setting for, 164
Plea for broader tendencies in veterinary instruction, a, 521
Pneumonia, antifebrine in infectious, 687
Pneumonomycosis due to the aspergillus fumigatus, a case of, 451
Post-mortem inspection of cattle, 217
  meat-inspection of swine, 75
Potassium chlorate for burns, 231
Powell, Edgar W., 220
Practical antisepsis in surgery, 615
Prevention of surgical shock, 689
Proceedings, A.V.M.A. 1899, new publications, 176
Production of camphor, 48[illegible]
Prolapsus of the uterus in a bitch, 95
    vaginæ in the bitch, 41[illegible]
Promotions of veterinarians in the Bureau of Animal Industry, 774
Prophylactic, antitetanic serum as a, 295
Proposed amendment to Constitution and By-laws A.V M.A., 511
Psoroptic scabies of cattle in Montana, 583
Publications, new—
  American Veterinary Medical Association, Proceedings, 1899, 176
  Antiseptic Treatment of Wounds, 48

Publications, new—
Diseases of Poultry, 177
Fourth revised edition of Gould, the, 176
Guide to Practical Meat Inspection, 49
Hobday's Canine and Feline Surgery, 701
Manual of Prescription Writing and Dosology, 49

QUININE and the malarial parasite, 231

RABIES and hydrophobia a rest, give, 359
and hydrophobia, 597, 663
in the bovine species, a prodromic symptom of, 158
a report of a case of, 93
the rapid diagnosis of, 404
spurious, 100
Rabiform symptoms due to spiroptera, 355
Ranck, E. M., V.M.D., 56, 252, 382
Ravenel, Mazyck P., M.D., 158, 228, 264, 404, 451
Rayen, W. C., M.D.C., 134
Reaction in hog-cholera, Gruber's, 528
Recognition, a well deserved, 46
Relation of tuberculosis in domesticated animals and man, 465
Reports of cases—
Actinomycosis and strongylus gigas in dogs, 421
Antitoxin treatment of distemper in horses, the, 171
Case reports, 39
Cases, some everyday, 37
Castration of an elephant, 478
Chicken, a case of aneurism in, 228
Cow, digestive stasis of the bowels in the, 342
Fistulæ, a new treatment for, 341
Fœtal skeleton, retention of, 761
Hogs, yellow, 480
Ilio-rectal fistula, 227
Jaundice, verminous, 480
Open joint where fluid had escaped, cure of a case of, 96
Paint (lead-poisoning) in cows, 344
Parturient apoplexy under Schmidt's treatment, 286
paresis, Schmidt's treatment of, 168
Prolapsus of the uterus in the bitch, 95
Rabies, reports of a case of, 93
Spasm of the masseters with complications, 169
Strongylus contortus in a herd of Angora goats, 423
Tendo-vaginitis of the metacarpo-phalangeal sheath, 344
Verminous ophthalmia (intra-ocular filariasis), 172
Report of Committee on Army Legislation, 569
Repp, John J., V.M.D., 646
Resection of the spinal accessory nerve for cribbing and roaring, 99
Retention of fœtal skeleton, 761
Review of contagious abortion, 151
Revision of the United States Pharmacopœia, 161
Reynolds, M. H., D.V.M., 27, 155, 268, 333
Rheumatism in domestic animals, 65
(Ridglings) castration of cryptorchids, 470
Rise of the veterinary profession, the, 20
Roger, H., 353
Rôle of iron in forming blood, the, 162
Routine manipulations and operations from the stand-point of asepsis and antisepsis, 1, 82
Russian work in veterinary science, some recent, 80

SAILER, JOSEPH, M.D., 228
Salley, I. L., 61, 189
Salmon, D. E., D.V.M., 177, 571, 597, 663
Salt an antidote to strychnine, 488
Salve, Lassar's sublimate-carbolic, 487
Sarcoptic scabies of the horse, 583
Saunders, Charles, V.S., 325
Schmidt's treatment of parturient paresis, 168,
apoplexy under, 286
Sciatic (femoro-popliteal) neurectomy, 98
Scott, T. W., V.S., 13
Selections, 159, 223, 488, 679
Serious charge, a, 234
Serum, antistreptococcic, 297
Serum as a prophylactic, antitetanic, 297
Shaw, Walter, 478
Sheath, tendo-vaginitis of the metacarpo phalangeal, 344
Shipping fever in horses, the antitoxin treatment of distemper or, 171
Shoulder, tumors on the, 743
Side lights of the Erie Convention, 646
Skin and hands, disinfection of the, 165
diseases, naftalan in obstinate, 166
Slaughter cure for tuberculosis, the, 679
Sloan, J. A., M.D.V., 35, 396, 416, 481, 547, 627, 682, 756
Smith, S. P., 151
Snake and insect bites, golindrinera, or euphorbia prostata for, 165
Soap, creolin, 488
Sodium bicarbonate for suppurating wounds, 166
Society Proceedings:
American Public Health Association, 514, 576
American Veterinary College, Alumni Association, 442
A. V. M. A., 179, 438, 633, 711, 775
Chicago Veterinary Society, 185, 251
French Veterinary Congress of 1900, 186
Genesee Valley Veterinary Medical Association, 183
Illinois State Veterinary Medical Association, 56, 711
Illinois Veterinary Medical and Surgical Association, 184, 573
Indiana Association of Veterinary Graduates, 120
Iowa State Veterinary Board, 442
Keystone Veterinary Medical Association, 55, 252
Maine Veterinary Medical Association, 61, 189
Maryland State Board of Veterinary Medical Examiners, 441
Massachusetts Veterinary Association, 118
Michigan State Board of Veterinary Medical Examiners, 441
Minnesota State Veterinary Medical Association, 123, 572
Missouri Valley Veterinary Association, 111, 179, 250, 440, 784
Missouri Veterinary Medical Association, 59, 575, 710, 712
New York State Veterinary Medical Society, 441
Niagara and Orleans County Veterinary Medical Society, 253, 514
North Dakota State Board of Veterinary Medical Examiners, 441
Ontario Veterinary Association, 109
Pennsylvania State Board of Veterinary Medical Examiners, 185
Pennsylvania State Veterinary Medical Association, 58, 107, 243, 382, 703, 779
South Dakota State Veterinary Medical Association, 709, 712
Veterinary Association of Manitoba, 188
Veterinary Medical Association of New Jersey, 121, 252, 381, 711, 712
Veterinary Medical Association of New York County, 317
Veterinary Medical Society, University of Pennsylvania, 60, 187

Society Proceedings:
Virginia State Board of Veterinary Medical Examiners, 441
Wisconsin's Society of Veterinary Graduates, 189, 249
Solutions of formaldehyde, 687
Some everyday cases, 337
of the dangers of an impure meat-supply and milk-supply, 260, 328
recent Russian work in veterinary science, 80
Somnifacients on the horse, the action of certain, 193, 278
South Dakota State Veterinary Medical Association, 709, 712
Spasm of the masseters, with complications, 169
Spider-webs as a hæmostatic, danger in using, 296
Spleen, hæmatoma of the, 292
Spurious rabies, 100
Stalker, M., 571
State control of glanders in Minnesota, 27
State work with infectious diseases of animals, 155, 268, 333
Sterilization of catgut by alcohol, 551
Stewart, S., sec., 440
Stomach-worms in lambs, 277
Strength, an added, 699
Strongylus contortus in a herd of Angora goats, 423
gigas in dogs, 421
Surgery of the penis, 481
practical antisepsis in, 615
Surgical shock, prevention of, 689
Stallions for the stud, examining and licensing of, 536
Strychnine, ancient use of, 551
salt as an antidote to, 488
Suture-wounds of the heart, 230
Surgery, practical antisepsis in, 615
Swain, S. H., V.S., 93, 227, 575
Swain, W. A., V.S., 185, 575
Sweetapple, C. H., 111
Swine. post-mortem inspection of, 75
Swiss pharmacopœia, the, 690
Syphilis to calves, an experiment in the transmission of, 264

Tannoform in diarrhœa, 420
Tendo-vaginitis of the metacarpo-phalangeal sheath, 344
Tennant, J. H., V.S., 286
Tetanus, 741
large doses of carbolic acid in equine, 624
Therapeutical and Pharmaceutical Notes:
Acetanilid in epilepsy, 690
Adulteration of yellow wax, 298
Aloes varieties, distinct therapeutic properties of, 688
Antifebrine in infectious pneumonia, 687
Antitetanic serum as a prophylactic, 297
Antiseptic wound oil, 487
Antistreptococcic serum, 297
Arsenic in organic bodies, 487
Bichloride eye wash, 487
Bladder any power of absorption, has the, 162
Boils, abortive treatment of, 690
Borax as a general poison antidote, 626
Boric acid, apomorphine counteracted by, 626
Camphorated oil a carbolic acid antidote, 167
Camphoroxol, 232
Carbolic acid, anthrax treated with hypodermatic injections of, 689
in equine tetanus, large doses of, 624
Chloretone, the effects and dangers of, 624
Therapeutical and Pharmaceutical Notes:
Cocaine, hypnotics antagonistic to, 624
Cocoa leaves, adulteration of, 295
Creolin soap, 488
Dehorning fluids, 164
Diarrhœa, tannoform in, 420
Diphtheria antitoxic globulins, 688
Discolored eserine solutions, 167
Disinfection of the skin and hands, 165
Dogs, acarus-mange in, 420
Epicarin, 752
Europhen as a surgical dressing, 551
Fluid extracts made with acetic acid, 689
tinctures made from, 163
Formaldehyde, solutions of, 687
Formula for the hypodermatic injection of acoin, 162
Gasoline, or petroleum ether, as a surgical detergent, 752
Gelatin, hæmostatic action of, 755
Goats, treatment of Basedow's disease by the milk of thyroidectomized, 419
Golindrinera, or euphorbia prostata, for snake and insect bites, 165
Harvey and the circulation of the blood, 298
Has the bladder any power of absorption? 162
Healing ointment, 487
powder, 551
Historical pharmacy, 298
Immunity of lower animals, 754
Inoculation against plague, 167
Iodine, age of tincture of, 295
Iron in forming blood, the rôle of, 162
Lassar's sublimate-carbolic salve, 487
Mange, treatment of equine, 753
Naftalan in obstinate skin diseases, 166
Oil of turpentine as a therapeutic agent, 485
Paraldehyde and chloroform for anæsthetic purposes, 623
Pharmacopœia, the Swiss, 690
Plaster-of-Paris, bard setting for, 164
Potassium chlorate for burns, 231
Poultices, use and abuse of, 295
Production of camphor, 486
Quinine and the malarial parasite, 231
Revision of the United States Pharmacopœia, 161
Salicylic acid in milk, detection of, 754
Salt an antidote to strychnine, 488
solution, a decinormal, 752
Soap, creolin, 488
Sodium bicarbonate for suppurating wounds, 166
Sodium bifluoride for histological preparations, 754
Spider webs as a hæmostatic, danger in using, 296
Spinal anæsthesia, danger in, 755
Sterilization of catgut by alcohol, 551
Strychnine, ancient use of, 551
Surgical shock, prevention of, 689
Suture-wounds of the heart, 230
Tetanus, large doses of carbolic acid in equine 624
Thyroid gland, action of the, 689
United States Dispensatory, 164
Urticaria, 753
Wounds, the immediate hermetic sealing of, 230
Zeroform in neurectomy, 418
Zinc, a plaster of oxide of, 419
Thirty-seventh Annual Convention, Detroit, September, 1900, 633, 775
Thomas, W. A., D.V.M., 671
Thorburn, W. W., 576
Thyroid gland, action of, 689
Thyroidectomized goats, treatment of Basedow's disease by the milk of, 419
Titus, H. E., D.V.M., 423
Torrance, F., sec., V.S., 189, 421

Tubercle bacilli, note on the examination of milk for, 395
Tuberculin test demanded, 238
in inter-State cattle traffic, obstacles to enforcing regulations requiring the, 590, 675
Tuberculosis, 473
among cattle at an altitude of 7400 feet, an outbreak of, 407
in domesticated animals and man, the relation of, 465
the slaughter cure for, 679
Tumors on the shoulder, 743
Turpentine as a therapeutic agent, oil of, 485

UNCLASSIFIED disease following parturition in cows, an, 24
United States College of Veterinary Surgeons, 648
Dispensatory, 164
Urethral calculi in the dog, 205
Urinary analysis in veterinary practice, 608
Urticaria, 753
Uterus in the bitch, prolapsus of the, 95

VANADIUM as a medicine, 232
Van Es, L., M.D., V.S., 344
Verminous jaundice, 480
ophthalmia, 172
Verschelden, O., V.S., 24
Veterinarian a factor in politics, the, 145
Veterinary Association of Manitoba, the, 188
corps, army, 361, 493, 552
instruction, a plea for broader tendencies in, 521
Medical Association of New Jersey, 121, 252. 381, 711, 712
Medical Association of New York County, 317
Veterinary medicine to public health, the relation of, 532
profession, rise of, 20
of the State of New Jersey, address to the, 148
progress in Michigan, 657
Vice-presidents, what shall we do with our, 562
Virginia State Board of Veterinary Medical Examiners, 441
Voorhees, Hon. Foster M., 712

WELCH, W. H., 761
Well-deserved recognition, 46
Wellner, Hermann, M. D., V.S., 65
Wheeler, A. S., V.M.D., 480
When will reason and judgment prevail? 695
Whitmore, N. P., M.D., 575
Wilcox, E. V., Ph.D., 80
Williams, Charles, V.M.D., 336
Wilson, Louis B., M.D., 722
Winchester, J. F., B.Sc., D.V.S., 649, 744
Wisconsin Society of Veterinary Graduates, 189, 249
Woman, passage of the bacillus of Koch into the milk of a tuberculous, 353
Worms, Albert C., sec., 58, 711
Wounds, healing of external, 396
the immediate hermetic sealing of, 230
Wright, J. B., Sec.-Treasurer, 251
Wyman, W. E. A., M.D.V., V.S., 35, 416, 481, 547, 627, 682, 737, 756

YELLOW hogs, 480
wax, adulteration of, 298

ZEROFORM in neurectomy, 418
Zinc, a plaster of oxide of, 419

